# Human Direct Skin Feeding Versus Membrane Feeding to Assess the Mosquitocidal Efficacy of High-Dose Ivermectin (IVERMAL Trial)

**DOI:** 10.1093/cid/ciy1063

**Published:** 2019-04-16

**Authors:** Menno R Smit, Eric O Ochomo, Ghaith Aljayyoussi, Titus K Kwambai, Bernard O Abong’o, Teun Bousema, David Waterhouse, Nabie M Bayoh, John E Gimnig, Aaron M Samuels, Meghna R Desai, Penelope A Phillips-Howard, Simon K Kariuki, Duolao Wang, Stephen A Ward, Feiko O ter Kuile

**Affiliations:** 1 Liverpool School of Tropical Medicine, United Kingdom; 2 Kenya Medical Research Institute, Centre for Global Health Research, Kisumu; 3 Kenya Ministry of Health, Kisumu County, Kisumu; 4 Radboud University Medical Center, Nijmegen, The Netherlands; 5 London School of Hygiene and Tropical Medicine, United Kingdom; 6 US Centers for Disease Control and Prevention, Center for Global Health, Division of Parasitic Diseases and Malaria, Atlanta, Georgia

**Keywords:** malaria, ivermectin, *Anopheles gambiae*, direct skin feeding, membrane feeding

## Abstract

**Background:**

Ivermectin is being considered for mass drug administration for malaria, due to its ability to kill mosquitoes feeding on recently treated individuals. In a recent trial, 3-day courses of 300 and 600 mcg/kg/day were shown to kill *Anopheles* mosquitoes for at least 28 days post-treatment when fed patients’ venous blood using membrane feeding assays. Direct skin feeding on humans may lead to higher mosquito mortality, as ivermectin capillary concentrations are higher. We compared mosquito mortality following direct skin and membrane feeding.

**Methods:**

We conducted a mosquito feeding study, nested within a randomized, double-blind, placebo-controlled trial of 141 adults with uncomplicated malaria in Kenya, comparing 3 days of ivermectin 300 mcg/kg/day, ivermectin 600 mcg/kg/day, or placebo, all co-administered with 3 days of dihydroartemisinin-piperaquine. On post-treatment day 7, direct skin and membrane feeding assays were conducted using laboratory-reared *Anopheles gambiae* sensu stricto. Mosquito survival was assessed daily for 28 days post-feeding.

**Results:**

Between July 20, 2015, and May 7, 2016, 69 of 141 patients participated in both direct skin and membrane feeding (placebo, n = 23; 300 mcg/kg/day, n = 24; 600 mcg/kg/day, n = 22). The 14-day post-feeding mortality for mosquitoes fed 7 days post-treatment on blood from pooled patients in both ivermectin arms was similar with direct skin feeding (mosquitoes observed, n = 2941) versus membrane feeding (mosquitoes observed, n = 7380): cumulative mortality (risk ratio 0.99, 95% confidence interval [CI] 0.95–1.03, *P* = .69) and survival time (hazard ratio 0.96, 95% CI 0.91–1.02, *P* = .19). Results were consistent by sex, by body mass index, and across the range of ivermectin capillary concentrations studied (0.72–73.9 ng/mL).

**Conclusions:**

Direct skin feeding and membrane feeding on day 7 resulted in similar mosquitocidal effects of ivermectin across a wide range of drug concentrations, suggesting that the mosquitocidal effects seen with membrane feeding accurately reflect those of natural biting. Membrane feeding, which is more patient friendly and ethically acceptable, can likely reliably be used to assess ivermectin’s mosquitocidal efficacy.

**Clinical Trials Registration:**

NCT02511353.

## Teaser

Ivermectin is being considered for mass drug administration for malaria, due to its ability to kill mosquitoes feeding on recently-treated individuals. Membrane feeding, which is more patient friendly, likely reflects the effects of direct-skin feeding in assessing ivermectin’s mosquitocidal efficacy.

Mass drug administration (MDA) for malaria is the treatment of the entire eligible population in an endemic area, regardless of individuals’ infection statuses or whether they have symptoms, and is currently being evaluated in several countries to accelerate progress towards malaria transmission reduction and elimination [1–3]. The antimalarial dihydroartemisinin-piperaquine (DP) is most commonly used for MDA, because of its slow elimination, providing 4–6 weeks of post-treatment prophylaxis against new infections. Ivermectin is an antiparasitic drug, which also kills mosquitoes feeding on recently treated individuals. Adding ivermectin to DP has been proposed as an innovative tool to increase the impact of MDA for malaria, by killing mosquitoes before they become infective (10–14 days after ingesting malaria parasites) and by reducing overall mosquito numbers in the community [4–6]. However, the single-dose of 150–200 mcg/kg ivermectin used for onchocerciasis and lymphatic filariasis control has only a small and short-lived effect (<7 days) on mosquito mortality [5]. Ivermectin is documented to be remarkably well tolerated, even up to doses of 2000 mcg/kg [7, 8].

In a recent trial, high-dose, 3-day courses of ivermectin at 300 and 600 mcg/kg/day, co-administered with DP, were shown to kill *Anopheles* mosquitoes for at least 28 days post-treatment when fed patients’ venous blood using membrane feeding assays [6]. Membrane feeding assays may, however, underestimate the mosquitocidal effect of ivermectin in comparison to direct skin feeding, where mosquitoes bite the human subject directly, due to potential differences in ivermectin concentrations between venous blood (used in membrane feeding) and blood in subdermal venules and arterioles (the main source of blood for mosquitoes during direct skin feeding). Ivermectin is known to accumulate in subcutaneous fat, dermal, and fascial tissue at 2- to 3-fold higher concentrations than in venous blood [9]. In the recent trial, ivermectin concentrations were 1.33-fold higher in capillary versus venous blood [10].

Ivermectin feeding studies with direct skin feeding on a human [11] and cattle [12] have shown a longer mosquitocidal effect (>2 weeks) in comparison with other studies using membrane feeding (<7 days) [13]. A single study, including 6 human subjects, compared direct skin and membrane feeding 4 hours after a single dose of ivermectin at 200 mcg/kg and found that mosquito mortality was higher after direct skin feeding (hazard ratio [HR] 1.73, 95% confidence interval [CI] 1.57–1.90, *P* = .0001) [14]. Any difference between feeding methods could have important implications for both pharmacokinetic-pharmacodynamic [10] and population-level [15] models assessing the impact of ivermectin on mosquito mortality and, through MDA, on malaria transmission. To date, these models have relied on membrane feeding estimates of ivermectin’s mosquitocidal efficacy.

We directly compared mosquito mortality following direct skin feeding versus membrane feeding in our trial on 3-day ivermectin courses of 300 and 600 mcg/kg/day.

## METHODS

### Trial Design

Details of the trial design, procedures, and safety, efficacy, and pharmacokinetic-pharmacodynamic (PK-PD) results have been published elsewhere [5, 6, 10]. Briefly, the study was a randomized, double-blind, placebo-controlled, parallel 3-arm, superiority trial (ClinicalTrials.gov: NCT02511353). Adults with uncomplicated *Plasmodium falciparum* malaria in western Kenya (n = 141) were randomly assigned (1:1:1), stratified by sex and body mass index (BMI), to receive 3 days of ivermectin 600 mcg/kg/day (n = 47), 300 mcg/kg/day (n = 48), or placebo (n = 46), all co-administered with 3 days of DP. The primary methodology used membrane feeding to assess the mosquitocidal efficacy of ivermectin. On day 7, the current nested sub-study compared direct skin feeding versus membrane feeding.

### Patients

All patients enrolled in the main trial were eligible to participate in the current sub-study, provided they gave additional written consent for direct skin feeding [5]. Up until day 7, when direct skin feeding took place, participants were given the opportunity to ask questions, familiarize themselves with the procedures in the lab, make their decision, and/or change their minds. Participation or refusal to participate in the direct skin feeding sub-study did not affect patients’ participation in the main trial or their malaria treatment. After direct skin feeding, patients were provided a tube of hydrocortisone cream to take home to reduce possible itching.

### Membrane Feeding Procedure

In accordance with a standard membrane feeding protocol [16], a 1 mL sample of the participant’s venous blood was drawn into a sodium-heparin–coated tube, preheated to 37.5°C. Within 2 minutes, the blood was placed in a water-jacketed glass-bell parafilm membrane feeding system, heated to 37.5°C, and 3 cups of mosquitoes commenced feeding for 20 minutes. The follow-up lasted 28 days for mosquito survival (2 cups) and 10 days for oocyst prevalence (1 cup).

### Direct Skin Feeding Procedure

Immediately after the blood draw for membrane feeding, and in accordance with previous direct skin feeding studies examining infectivity [17], 1 cup of mosquitoes was placed directly on the skin of the participant and allowed to feed for 15 minutes. The follow-up lasted 28 days for mosquito survival.

### General Insectary Procedures

For both methods, each feeding used new cups of 50, 3- to 5-day-old, female, insectary-reared, infection-free, *Anopheles gambiae* sensu stricto. Kisumu-strain mosquitoes. Post-feeding, the number of mosquitoes with an engorged abdomen (fully fed) were counted and those with lean abdomens (semi- and unfed) were discarded. Each day, the number of dead mosquitoes were counted and removed until the end of the follow-up period (see feeding procedures above). After the initial feeding on human blood, the mosquitoes were kept in a temperature- and humidity-controlled insectary (27°C, 80%) with a fixed light-dark cycle (12h/12h) and were maintained *ad libitum* on 10% sugar feeds. Insectary staff assessing mosquito survival were blinded to all characteristics of the cups, including participant identification, study arm, duration between treatment and feeding, and feeding method.

### Outcome Measures

The primary outcome was cumulative mosquito mortality 14 days after feeding (henceforth referred to as post-feeding) on blood taken from patients who had started the 3-day ivermectin and DP regimen 7 days earlier (henceforth referred to as post-treatment). The secondary outcome was the daily survival of mosquitoes up to day 14 post-feeding. Paired venous and capillary ivermectin plasma concentrations were collected on post-treatment days 2–7; The predicted concentrations on day 7 were obtained from our previously-published PK-PD analysis [10].

### Statistical Analysis

The analysis was based on the intention-to-treat population. Only mosquito and pharmacokinetic data from participants that contributed to both direct skin and membrane feeding was included. Mosquito mortality was assessed for fully-fed mosquitoes. Cumulative mosquito mortality was analyzed using the generalized estimating equations (GEE) model, with a binomial distribution, log link function, the feeding method (direct skin or membrane) as the only predictor, and taking the cluster design into account. Risk ratios (RR) and their 95% confidence intervals were derived from the GEE model. The survival time of mosquitoes post-feeding was analyzed using Cox regression, with feeding method as the only predictor, and adjusted for mosquito clusters (using shared frailty with γ distribution) to derive HRs. For GEE models, analyses were based on data collected for approximately 100 mosquitoes (2 cups of 50) per participant for membrane feeding and 50 mosquitoes (1 cup of 50) per participant for direct skin feeding. Additional membrane-fed mosquitoes used for oocyst polymerase chain reaction (1 cup of 50) were excluded from the GEE analyses on day 14, as they had all been euthanized after 10 days of mosquito follow-up. For Cox models, analyses were based on data collected from approximately 150 mosquitoes (3 cups of 50) per participant for membrane feeding and 50 mosquitoes (1 cup of 50) per participant for direct skin feeding. This included the membrane-fed mosquitoes used for oocyst polymerase chain reaction (1 cup of 50), which were euthanized after 10 days and, therefore, contributed a maximum of 10 days of survival data. The above analyses were performed separately by treatment arm and pooled across the 2 ivermectin arms. Pearson’s correlation coefficients (ρ) were determined for mosquito mortality rate ratios (direct skin versus membrane feeding) and ivermectin concentrations (capillary-venous ratios), and were stratified by known determinants of ivermectin pharmacokinetics (sex and BMI) [10]. Analyses were performed using Stata v14.2.

### Ethics

All patients gave written informed consent to participate in the main trial and additional written informed consent to participate in direct skin feeding on day 7 post-treatment. The study was approved by the ethics committees of the participating institutions [5].

## RESULTS

**Figure 1.**
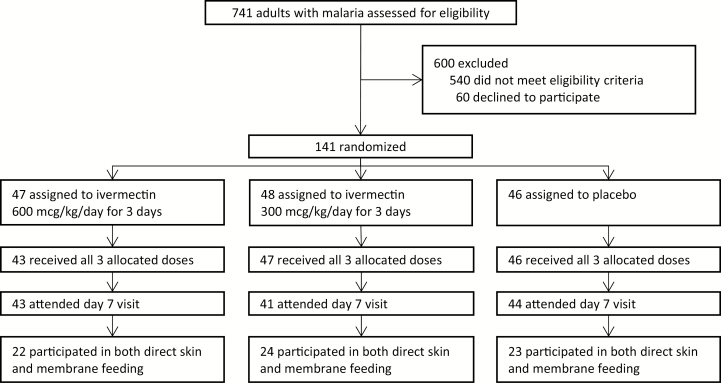
Trial flowchart.

Between July 20, 2015, and May 7, 2016, 141 patients were randomized to ivermectin 600 mcg/kg/day (n = 47), 300 mcg/kg/day (n = 48), or placebo (n = 46). In total, 128 patients (90.8%) attended the primary outcome visit at 7 days post-treatment, of which 69 patients (54%) participated in both direct skin and membrane feeding (ivermectin 600 mcg/kg/day, n = 22; ivermectin 300 mcg/kg/day, n = 24; placebo, n = 23; Figure 1; Table 1). All patients were treated per protocol, except two in the 600 mcg/kg/day arm.

**Table 1.** Characteristics of Subjects That Participated in Both Direct-skin and Membrane Feeding

	Ivermectin 600 mcg/kg/day for 3 Days (n = 22)	Ivermectin 300 mcg/kg/day for 3 Days (n = 24)	Placebo (n = 23)
Age, years	27.3 (7.4)	25.5 (7.5)	26.0 (5.0)
Sex			
Male	13 (59%)	16 (67%)	14 (61%)
Female	9 (41%)	8 (33%)	9 (39%)
Body mass index, kg/m^2^	22.9 (3.4)	21.5 (3.0)	21.6 (2.6)

Data are shown as n (%) or mean (standard deviation). Baseline characteristics of subjects that participated in both direct skin and membrane feeding (n** = **69) were similar to those of the other trial participants that did not (n** = **72) [6].

The proportion of mosquitoes that fully fed was higher for direct skin feeding (2941/3446, 85.3%) versus membrane feeding (7380/10 368, 71.2%; RR 1.20, 95% CI 1.12–1.28; *P* < .0001); this did not differ by treatment arm for either method (Table 2).

**Table 2.** Proportion of Fully Fed Mosquitoes After Direct Skin Feeding and Membrane Feeding

Feeding Method	Human Subjects; Mosquitoes Fully Fed (%)^a^						Risk Ratio (95% Confidence Interval), *P* Value		
	IVM-3x600		IVM-3x300		Placebo		IVM-3x600 vs Placebo	IVM-3x300 vs Placebo	IVM-3x600 vs IVM-3x300
Direct skin	22;	938/1096 (85.6)	24;	1015/1199 (84.7)	23;	988/1151 (85.8)	1.00 (0.89–1.11), .95	0.99 (0.88–1.10), .80	1.01 (0.91–1.12), .84
Membrane	22;	2584/3300 (78.3)	24;	2533/3613 (70.1)	23;	2263/3455 (65.5)	1.20 (0.99–1.45), .07	1.07 (0.88–1.30), .51	1.12 (0.94–1.33), .20

Abbreviations: IVM-3x300**, **ivermectin 300 mcg/kg/day for 3 days; IVM-3x600**, **ivermectin 600 mcg/kg/day for 3 days.

^a^The number of mosquitoes fully fed out of the number of mosquitoes offered a blood meal.

**Figure 2.**
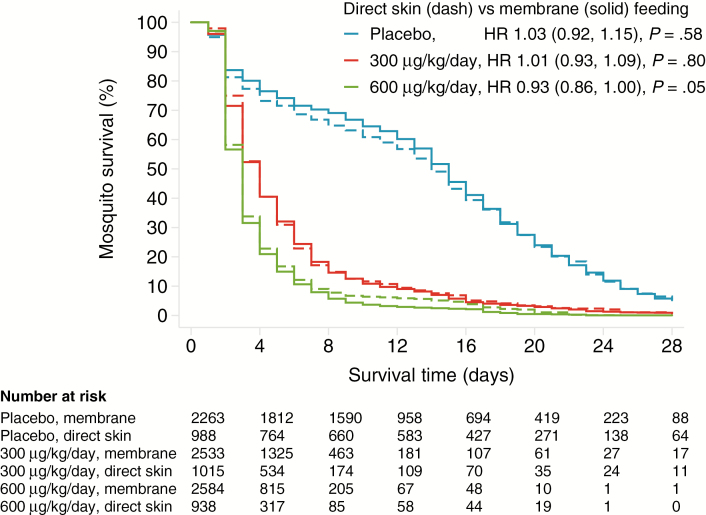
Mosquito mortality, stratified by treatment arm and feeding method. Direct skin feeding is indicated by the dashed lines; membrane feeding is indicated by the solid lines. Ivermectin 600 mcg/kg/day is indicated by the green lines; ivermectin 300 mcg/kg/day is indicated by the red lines; and placebo is indicated by the blue lines. Mortality was measured following feeding on day 7 post-treatment. The hazard ratios (95% confidence interval, *P* value) of mortality during the 14 days post-feeding, comparing direct skin versus membrane feeding for each treatment arm, were adjusted for mosquito clusters. Abbreviation: HR, hazard ratio.

Compared with membrane feeding, direct skin feeding was associated with similar 14-day post-feeding mosquito mortality when fed on blood 7 days post-treatment, both in terms of cumulative mortality (ivermectin 600 mcg/kg/day, RR 0.98, 95% CI 0.90–1.06, *P* = .55; ivermectin 300 mcg/kg/day, RR 1.01, 0.98–1.03, *P* = .69; placebo RR 1.07, 0.88–1.29, *P* = .51) and survival time (ivermectin 600 mcg/kg/day, HR 0.93, 0.86–1.00, *P* = .05; ivermectin 300 mcg/kg/day, HR 1.01, 0.93–1.09, *P* = .80; placebo HR 1.03, 0.92–1.15, *P* = .58; Figure 2; Table 3). Similar results were seen upon pooling the two ivermectin arms (HR 0.96, 0.91–1.02, *P* = .19; RR 0.99, 0.95–1.03, *P* = .69).

**Table 3.** Mosquito Mortality Following Direct Skin Feeding and Membrane Feeding

Treatment Group	Human Subjects	Mosquito Mortality on Day 14 (%)		Risk or Hazard Ratio (95% CI), *P* Value	
		Direct Skin Feeding	Membrane Feeding^a^	Model	Direct Skin vs Membrane
IVM-3x600	22	890/938 (94.9)	1677/1729 (97.0)	GEE	RR 0.98 (0.90–1.06), .55
			2514/2584 (97.3)	Cox	HR 0.93 (0.86–1.00), .052
IVM-3x300	24	938/1015 (92.4)	1573/1703 (92.4)	GEE	RR 1.01 (0.98–1.03), .69
			2330/2533 (92.0)	Cox	HR 1.01 (0.93–1.09), .80
Placebo	23	503/988 (50.9)	706/1493 (47.3)	GEE	RR 1.07 (0.88–1.29), .51
			999/2263 (44.1)	Cox	HR 1.03 (0.92–1.15), .58

Abbreviations: CI, confidence interval; GEE, generalized estimating equations; HR**, **hazard ratio; IVM-3x300**, **ivermectin 300 mcg/kg/day for 3 days; IVM-3x600**, **ivermectin 600 mcg/kg/day for 3 days; RR, risk ratio.

^a^GEE models used 2 cups of mosquitoes, followed for 14 days; Cox models used the same 2 cups, plus 1 cup, followed for 10 days, which were then euthanized for oocyst polymerase chain reaction.

**Figure 3.**
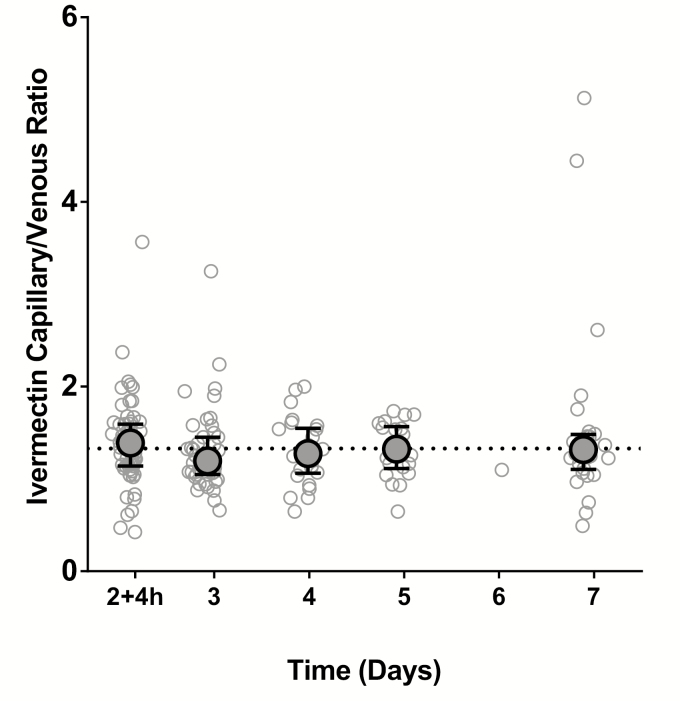
Capillary versus venous ratios of ivermectin plasma concentration during 2–7 days post-treatment. The open circles represent the capillary versus venous ratios of observed ivermectin plasma concentrations for each sample (n** = **177), taken from patients in the main trial contributing capillary samples (n** = **61) during 2–7 days post-treatment (maximum 4 samples/patient). The ball-whiskers indicate the median ± interquartile range per sampling day. The horizonal line indicates a median ratio of 1.33 (5th–95th percentiles, 0.98–1.63), based on the trial’s simultaneous pharmacokinetic-pharmacodynamic population model [10]. Adapted from Smit et al [10].

Based on the previously published trial’s population PK-PD model [10], the predicted medians (5th–95th percentiles [p5–p95]) of ivermectin concentrations in venous blood at day 7 were 17.3 (2.87–43.0), 7.75 (1.58–18.7), and 11.3 (1.58–39.5) ng/mL for ivermectin 600 mcg/kg/day, 300 mcg/kg/day, and in the arms combined, respectively. The corresponding predicted capillary blood concentrations were 24.2 (6.04–58.6), 8.22 (1.54–22.6), and 14.3 (p5–p95, 1.60–49.4; min–max 0.72–73.9) ng/mL, respectively. The capillary-venous ratio of the observed ivermectin plasma concentrations remained consistent from day 2 + 4 hours through day 7, near the population predicted median ratio of 1.33 (Figure 3) [10].

**Figure 4.**
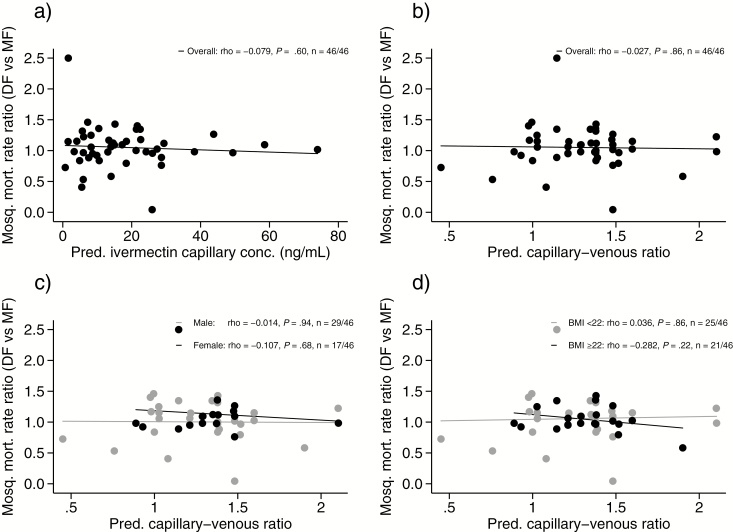
Direct skin feeding versus membrane feeding ratios of mosquito mortality rates by ivermectin concentration and capillary-venous ratio at the time of feeding. The circles represent the observed 14-day mosquito mortality rate ratios of direct skin versus membrane feeding, performed at day 7 post-treatment for each patient that received ivermectin and consented to direct skin feeding (n** = **46), plotted against their day 7 (*A*) predicted ivermectin capillary plasma concentration and (*B*) predicted capillary versus venous ratio, using the trial’s simultaneous pharmacokinetic-pharmacodynamic population model [10]. (*C*) and (*D*) are as per (*B*), but now stratified by sex and body mass index, respectively. The lines indicate the linear fits. Abbreviation: BMI, body mass index; DF, direct skin feeding; MF, membrane feeding; mosq., mosquito; mort., mortality; pred., predicted.

The median (p5–p95) mosquito mortality rates (deaths/100 days) per sample for each feeding method (both ivermectin arms pooled) at day 7 of the study were 24.1 (6.96–50.0) for direct skin feeding and 24.0 (6.73–48.3) for membrane feeding. The ratio of direct skin versus membrane feeding mosquito mortality rates was not affected by patients’ ivermectin plasma concentrations or capillary-venous ratios within the ranges studied, either overall or when stratified by sex and BMI (Figure 4).

## DISCUSSION

Direct skin feeding and membrane feeding conducted at day 7 post-treatment resulted in similar mosquitocidal effects of ivermectin. This was seen in each of the 300 and 600 mcg/kg/day treatment arms separately and when combined, was not dependent on patients’ ivermectin plasma concentrations or capillary-venous ratio, and was seen irrespective of whether mortality was assessed as a proportion or a rate. Membrane feeding, which is more patient friendly, can likely reliably be used to assess ivermectin’s mosquitocidal effects.

Although mosquito mortality was only assessed at a single time point post-treatment, on day 7, results may be applicable to earlier or later feeding time points. This is because the lack of difference in mosquito mortality between the 2 feeding methods was observed across the full range of ivermectin capillary concentrations tested (min–max, 0.72–73.9 ng/mL; Figure 4), with corresponding mosquito mortality rates (direct skin feeding: median 24.1 deaths/100 days, p5–p95 6.96–50.0; membrane feeding: median 24.0, p5–p95 6.73–48.3) covering nearly the entire mosquitocidal effect range found in the main trial (median effect E_50_ 28.7 deaths/100 days, E_min_-E_max_ 3.9–53.4) [10]. Although differences between direct skin and membrane feeding were not assessed for capillary concentrations above 73.9 ng/mL, the mosquito mortality by day 14 at these concentrations (incidence rate ratio > 10.6) is near universal, making it unlikely that clinically meaningful differences would exist between feeding approaches. Although it is possible that a differential effect between direct skin and membrane feeding is only evident at lower concentrations, when the mosquitocidal effect is low, this is not suggested by our analyses, which show a similar lack of difference between the 2 feeding methods even at the lowest concentrations studied.

It is unclear why the higher concentration of ivermectin in capillary blood compared to venous blood—a capillary-venous plasma ratio of 1.33, which was consistent across the range of blood concentrations tested in the main trial (Figure 3) [10] does not translate to higher mosquito mortality in direct skin feeding. The surface area available for feeding was larger for direct skin feeding (8 cm diameter of the cup exposed to the skin vs 1.8 cm diameter of the artificial membrane), possibly leading to less crowding and explaining the higher proportion of fully-fed mosquitoes in direct skin feeding. If capillary blood samples reflect the blood source of skin-fed mosquitoes and the concentration of ivermectin imbibed, counterbalancing forces must be at play. A possible explanation is that direct skin fed mosquitoes consumed a smaller blood volume than membrane fed mosquitoes, despite all analyzed mosquitoes visually appearing to be fully fed. This was suggested in a previous study with *Anopheles aquasalis*, a Latin American malaria vector, that found a 48% difference in mean post-feeding weight between direct skin fed (0.040 mg, standard deviation 0.02) and membrane fed mosquitoes (0.059, standard deviation 0.02) [14]. Such a difference in blood-meal size may reflect differences in the blood flow between the 2 procedures or the energy involved in taking a blood meal, both of which might favor a larger blood meal in membrane feeding. Future studies could assess blood-meal volumes following direct skin and membrane feeding, for example by measuring the hemoglobin in fed mosquitoes. Furthermore, it is not clear whether other factors associated with skin feeding that are not present in the membrane feeds (eg, dermal immune mechanisms) could reduce ivermectin’s mosquitocidal effect with direct skin feeding.

Only one previous study has directly compared direct skin versus membrane feeding [14]. This small study in 6 human subjects in Brazil used feeding assays conducted 4 hours after a single-dose of 200 mcg/kg (ie, T_max_, time of the maximum drug concentration in serum) and reported significantly higher mortality of the Latin American malaria vector *Anopheles aquasalis* following direct skin feeding [14]. A single dose of ivermectin at 200 mcg/kg has a predicted plasma maximum drug concentration (median, p5–p95) of 27 ng/mL (18.8–41.4) [5], which is within the 1.58–39.5 ng/mL range of the venous plasma concentrations tested in our current study. The HR following membrane feeding at 4 hours after this single 200 mcg/kg dose was 3.2, which is not that different from the 4.4 in the 300 mcg/kg/day arm in our study. It is not clear whether the differences between the 2 studies can be explained by differences in pharmacodynamic factors (ie, ivermectin sensitivity of the *Anopheles* species) or pharmacokinetic factors, due to differences in study populations (ie, ethnic group and clinical indication; Kenyan patients with acute uncomplicated malaria versus Brazilian patients with other indications for ivermectin treatment) or differences in the timing of feeding post-treatment (ie, 7 days versus 4 hours after ingestion). It is also possible that the higher mosquito mortality observed with direct skin feeding in the Brazilian study reflects a chance finding, given the small number of subjects (n = 6).

As the relationship between ivermectin concentration, whether venous or capillary, and mosquitocidal effect has been previously established for membrane feeding [10] and the current study shows no difference in mosquitocidal efficacy between direct skin and membrane feeding with *Anopheles Gambiae ss*, future studies could consider using ivermectin concentration in either venous blood or in capillary blood obtained from finger-prick samples as a proxy of the potential mosquitocidal effect, without the need to invoke more labor-intensive and patient-unfriendly membrane or direct skin feeding assays. The similarity in mosquitocidal efficacy between feeding methods also has important implications for population-level models used to predict the impact of ivermectin MDA on malaria transmission [15]. Due to the sparse availability of direct skin feeding data, these models have relied on mosquitocidal efficacy estimates from membrane feeding, using either spiked blood [12, 13, 18, 19] or blood samples from humans [13]. Our results, which show that membrane feeding appears to be a good proxy for natural biting, strengthen the reliability of these existing models.

Our current nested sub-study was limited by the fact that it was only conducted at a single time point, at day 7 post-treatment. Future studies could examine differences between direct skin and membrane feeding at lower and higher concentrations, at earlier or later time points post-treatment, and using both ivermectin and other endectocides, such as moxidectin, eprinomectin, fluralaner, and afoxolaner [20–22], which have different pharmacokinetics. Furthermore, the trial only assessed mosquito mortality and did not assess any possible sublethal effects, such as sporogony and oviposition (laying of eggs), which could be relevant, especially at low concentrations, and could be investigated in further studies. It is unknown whether our results can be extrapolated to the pediatric population, because ivermectin pharmacokinetics in children, including the capillary-venous ratio, are not yet known.

In conclusion, both direct skin feeding and membrane feeding on day 7 resulted in similar mosquito mortality of *Anopheles gambiae* after ivermectin treatment across a wide range of drug concentrations; this was similar by sex and BMI, suggesting that the mosquitocidal effects observed with membrane feeding in the main trial depict those of natural biting. Membrane feeding, which is more patient-friendly and allows a larger number of mosquito observations, likely accurately reflects ivermectin’s mosquitocidal effects.

## Notes

***Author contributions.*** F. O. tK. and M. R. S. conceived the study. M. R. S., P. A. P.-H., and F. O. tK. wrote the grant. M. R. S., E. O. O., and F. O. tK. drafted the protocol. All investigators contributed to its refinement and approved the final version, except for D. Wat., who joined later. D. Wang was the trial statistician. G. A. and S. A. W. conducted the Monte Carlo simulations to define the dosing regimens and further developed the pharmacokinetic sub-studies. M. R. S., E. O. O., T. K. K., and B. O. A. performed the field work. D. Wat. and S. A. W. developed, validated, and carried out the drug analytical quantification. M. R. S. and D. Wang analyzed the data. M. R. S. and F. O. tK. drafted the manuscript. All authors contributed to and approved the final manuscript prior to submission.

***Acknowledgments.*** The authors thank all those that made this study possible, including the study participants and staff, the members of the Trial Steering Committee (Teun Bousema, Kevin Kobylinski, and Brian Foy), and the members of the Data Monitoring & Ethics Committee (Alejandro Krolewiecki, James Oloo, Timothy Collier, and Carlos Chaccour). They thank the Mundo Sano Foundation and Elea Laboratories for donating the ivermectin. Finally, they thank the Jaramogi Oginga Odinga Teaching and Referral Hospital and the Kisumu County Ministry of Health for hosting the study.

***Financial support.*** This work was supported by the Malaria Eradication Scientific Alliance, through a sub-grant from the Bill and Melinda Gates Foundation. Co-funding was provided by the US Centers for Disease Control and Prevention, through a Cooperative Agreement between the Centers for Disease Control and Prevention and the Liverpool School of Tropical Medicine.

***Potential conflicts of interest.*** All authors: No reported conflicts. All authors have submitted the ICMJE Form for Disclosure of Potential Conflicts of Interest. Conflicts that the editors consider relevant to the content of the manuscript have been disclosed.
